# Harnessing Polyethylene Glycol 3350 for Enhanced Peripheral Nerve Repair: A Path to Accelerated Recovery

**DOI:** 10.3390/medicina61040624

**Published:** 2025-03-28

**Authors:** Erdinç Tunç, Ejder Saylav Bora, Oytun Erbaş

**Affiliations:** 1 Faculty of Medicine, Department of Anatomy, Biruni University, 34015 Istanbul, Türkiye; etunc@yahoo.com; 2 Faculty of Medicine, Department of Emergency Medicine, Izmir Katip Çelebi University, 35620 Izmir, Türkiye; 3 Faculty of Medicine, Biruni Research Center (BAMER), Biruni University, 34015 Istanbul, Türkiye; oytunerbas2012@gmail.com

**Keywords:** polyethylene glycol, nerve injury, electromyography

## Abstract

*Background and Objectives:* Peripheral nerve injuries often result in significant functional impairment, and complete recovery remains challenging despite surgical interventions. Polyethylene glycol (PEG) has shown promise in nerve repair by facilitating axonal fusion and inhibiting Wallerian degeneration. This study investigates the biochemical, histopathological, and electrophysiological effects of PEG 3350 in a sciatic nerve injury model. *Materials and Methods*: Thirty adult male Wistar rats were divided into three groups: a control group, a surgery and saline group, and a surgery and PEG 3350 treatment group. Sciatic nerve transection was performed, and PEG 3350 was administered intraperitoneally for 12 weeks. Electromyography (EMG) and the inclined plane test assessed functional recovery. Sciatic nerve tissues were analyzed histologically and biochemically, including nerve growth factor (NGF), heat shock protein 70 (HSP-70), and malondialdehyde (MDA) levels. *Results*: PEG 3350 significantly improved electrophysiological parameters, reducing compound muscle action potential (CMAP) latency and increasing CMAP amplitude compared to the saline group (*p* < 0.05). Functional recovery, assessed by the inclined plane test, showed a significant improvement in the PEG-treated group (*p* < 0.01). Biochemical analysis revealed increased NGF and HSP-70 levels, suggesting enhanced neuroprotection and regeneration. Histopathological analysis demonstrated reduced fibrosis and increased axonal density in the PEG group compared to controls. PEG 3350 enhances nerve regeneration by improving electrophysiological function, promoting axonal repair, and increasing neurotrophic factor expression. *Conclusions*: These findings suggest PEG as a potential adjunct therapy for peripheral nerve injuries. Future research should explore the optimal administration protocols and combined therapeutic strategies for maximizing recovery.

## 1. Introduction

Peripheral nerve injuries pose a considerable clinical problem, frequently resulting in chronic pain, sensory impairments, and motor dysfunction, significantly affecting the quality of life for those impacted [1]. While the peripheral nervous system possesses an inherent capacity for regeneration following injury, complete functional restoration remains elusive in many cases [1,2]. Despite advancements in microsurgical techniques aimed at expediting the recovery process, full recovery is still uncommon, particularly when nerve injury is compounded by polytrauma, necessitating delayed surgical intervention [2]. Given the limitations of surgical interventions and the often incomplete functional recovery, there is a pressing need for adjunctive therapies that can promote nerve regeneration and improve patient outcomes [3,4].

The recovery is primarily affected by the degree, extent, kind of damage, the duration from injury to treatment, and the patient’s age [5]. These injuries are particularly challenging due to Wallerian degeneration, which significantly slows axonal regeneration to approximately 1 mm per day or just 1 mm per day following complete nerve division or neurotmesis [6,7]. Although it is known that micro-surgical intervention is the gold standard in peripheral nerve injury [8], it is still said that this procedure alone is insufficient. Studies are ongoing on whether an agent can be applied during the healing process or intraoperatively [5,9].

Effective imaging is crucial for rapid and effective nerve regeneration treatment. Recent advances in nerve regeneration research underscore the important role of imaging techniques in monitoring recovery processes. High-resolution ultrasound and magnetic resonance imaging (MRI) and MRI-derived 3D reconstructions and diffusion tensor imaging (DTI) have demonstrated efficacy in accurately identifying peripheral nerve injuries and informing surgical procedures [10,11]. These techniques improve diagnostic accuracy and facilitate better nerve regeneration and functional recovery assessment.

Polyethylene glycol (PEG) is a polymer with a wide range of applications, including its use in hydrogels for tissue culture, as a laxative for children [12], and in the modification of proteins [13]. PEG functions by merging disrupted axonal membranes, aiding in the restoration of axonal continuity and inhibiting Wallerian degeneration. This procedure facilitates the prompt restoration of action potentials at the damage site, enhancing nerve conduction and functional recovery [14,15,16]. PEG’s capacity to modify gene expression in brain and muscle contexts reinforces its restorative attributes, with notable differential gene regulation identified in treated patients. By encouraging the fusion of damaged axonal membranes, PEG functions as a fusogen, restoring axonal continuity and enabling the prompt restoration of nerve impulse conduction [17]. PEG helps maintain nerve function by preventing Wallerian degeneration, a process in which the portion of the axon detached from the neuron’s cell body degenerates [18].

In several recently published experimental studies, PEGs have demonstrated that they stabilize cell membranes, thereby reducing oxidative stress [19,20]. According to research, PEG indirectly lowers inflammation by reducing NF-κB and TNF-α levels based on its protective effect on cell membrane permeability [21,22]. Furthermore, in a different study, Chen et al. discovered that PEG improves overall neurological function by preventing direct mitochondrial damage and physically filling defective holes in the mitochondrial membrane [23].

PEG has exhibited advantages over conventional (Peripheral nerve repair and grafting techniques in surgery) nerve healing methods in both animal models and preliminary human studies. In animal experiments, PEG therapy improved electrophysiological, histological, and behavioral outcomes, with fast restoration of axonal integrity and increased recovery of motor function [15,16,24]. Clinical research indicates that PEG-fused nerves have markedly superior sensory recovery and enhancements in quality of life relative to controls, with no documented side effects [14,16].

Although PEG therapy has potential, other technical issues persist, including the ideal timing for administration following damage and the possibility of systemic or localized therapies enhancing its efficacy. In this study, we investigated the biochemical and histopathologic effects of PEG, which is known to be effective in nerve damage, on nerve tissue by supporting it with electromyography and power tests.

## 2. Materials and Methods

### 2.1. Animal Model

This research encompassed 30 adult male Wistar rats, each weighing between 200 and 210 g. The animals were kept in a controlled environment with a 22 ± 2 °C temperature and a 12-h light/dark cycle. During the study, they were granted unrestricted access to tap water and supplied with a standard pellet diet. Ethical approval was secured from the Animal Ethics Committee of the University of Science (Approval No: 2525023403). All reagents and chemicals were obtained from Merk Sigma-Aldrich, Burlington, MA, USA. unless stated otherwise.

### 2.2. Experimental Design

**Figure 1:**
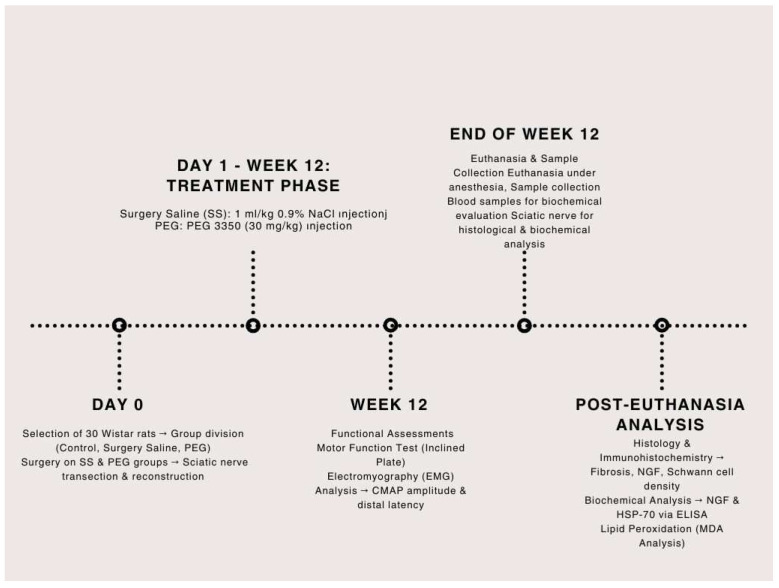
Experimental timeline diagram representing grouping, surgical intervention, treatment period, and final assessments.

A total of 30 male Wistar rats were chosen for this study. Of the rats, 20 underwent surgical procedures aimed at the sciatic nerve, while the remaining 10 constituted a control group, receiving no surgical intervention or pharmacological treatment. The experimental group was subdivided into two subgroups: the Surgery and Saline (SS) group (n = 10), which received an intraperitoneal injection of 1 mL/kg 0.9% NaCl, and the Surgery and Polyethylene Glycol 3350 (PEG) group (SPEG) (n = 10), which was administered PEG 3350 (30 mg/kg, Merk Sigma-Aldrich, Burlington, MA, USA) intraperitoneally daily for 12 weeks (Figure 1).

After the treatment, motor function assessments were performed, and electromyographic (EMG) recordings were acquired to assess nerve regeneration. After the study, all animals were euthanized via cervical dislocation while under profound anesthesia induced by ketamine (100 mg/kg, Ketasol, Richterpharma AG, Wels, Austria) and xylazine (50 mg/kg, Rompun, Bayer, Germany). Sciatic nerve tissues were harvested for subsequent immunohistochemical and biochemical analyses.

### 2.3. Operative Intervention

Following the administration of intraperitoneal injection of ketamine (75 mg/kg) and xylazine (10 mg/kg) for anesthesia, the animals were positioned prone on a sterile surgical platform. Surgery was conducted under aseptic conditions using standard sterile techniques. Bilateral sciatic nerves were exposed roughly 1 cm distal to their trifurcation, with a 3–3.5 cm segment meticulously isolated from adjacent tissues. A nerve transection was executed using fine microsurgical scissors at a location 1.5 cm proximal to the trifurcation of the tibial, common peroneal, and caudal sural cutaneous nerves. A single surgeon subsequently executed microsurgical reconstruction utilizing three epi-neural sutures (Ethilon^®^ 9-0, Ethicon, Raritan, NJ, USA) to guarantee accurate realignment. The surgical incision was sutured with 3-0 Vicryl^®^ sutures. Post-surgery, the animals were returned to their enclosures and granted unrestricted access to food and water.

### 2.4. Electrophysiological Evaluations

Electromyographic (EMG) recordings were performed under anesthesia induced by ketamine (80 mg/kg) and xylazine (10 mg/kg) to assess nerve function. Bipolar subcutaneous needle electrodes (BIOPAC Systems, Inc., Santa Barbara, CA, USA) were positioned at the sciatic notch to administer supramaximal stimulation with the following parameters: 10 V intensity, 0.05 ms pulse duration, 1 Hz frequency, 0.5–5000 Hz bandwidth, and a 40 kHz/s sampling rate.

Electromyographic responses, comprising compound muscle action potentials (CMAPs), were obtained from the second and third interosseous muscles utilizing unipolar platinum electrodes. Data collection and analysis were conducted utilizing BIOPAC Student Lab Pro software (version 3.6.7, BIOPAC Systems, Inc.). The main parameters assessed were distal latency and compound muscle action potential (CMAP) amplitude. To maintain stability, rectal temperature was continuously monitored with a rectal probe (HP Viridia 24-C, Hewlett-Packard Company, Palo Alto, CA, USA) and regulated between 36 and 37 °C using a heating pad. All EMG evaluations were performed between 10:00 a.m. and 2:00 p.m.

### 2.5. Assessment of Motor Function Utilizing the Inclined Plate Technique

The inclined plate test was conducted to evaluate motor function. Each rat was placed along the longitudinal axis of an adjustable inclined platform, initially inclined at a 10-degree angle. The platform’s angle was progressively elevated until the rat could no longer sustain its position for at least 5 s without slipping. The maximum angle at which the rat maintained stability was documented as its motor performance score. Each animal participated in three trials, and the mean of these trials was utilized for analysis.

### 2.6. Histological and Immunohistochemical Analysis

Intracardiac perfusion with 4% formaldehyde was conducted for tissue analysis to preserve the sciatic nerve specimens. A 10-mm distal segment from the injury site was excised, preserved in formaldehyde, embedded in paraffin, and sectioned into 5-µm slices utilizing a microtome (Leica RM 2145, Nussloch, Germany). Hematoxylin and eosin staining were employed to elucidate nerve structures.

Fibrotic alterations were evaluated by enumerating cellular components in five randomly selected fields per sample. The degree of fibrosis was assessed by calculating the percentage of fibrotic area about the total field area. Immunohistochemical analysis entailed inhibiting endogenous peroxidase activity using 10% hydrogen peroxide (H_2_O_2_) for 30 min, succeeded by a one-hour incubation with normal goat serum (Invitrogen, Waltham, MA, USA) at ambient temperature. The sections were subsequently incubated overnight at 4 °C with a primary antibody targeting nerve growth factor (NGF) (1:100, Santa Cruz Biotechnology, Dallas, TX, USA). Detection was conducted utilizing a rabbit IgG-specific Histostain-Plus Bulk kit (Invitrogen), with visualization achieved through 3,3′-diaminobenzidine (DAB) staining. Microscopic imaging was performed with an Olympus BX51 microscope (Olympus Corp., Tokyo, Japan), and digital images were obtained using an Olympus C-5050 camera (Olympus Corp., Tokyo, Japan).

Quantitative immunohistochemical analysis was conducted on six sections per specimen, with immune-positive Schwann cells and axons enumerated at 20× magnification by two independent, blinded researchers. Measurements of axonal density and diameter were conducted in six randomly selected fields (one central and five peripheral) utilizing 20× magnification. For the histological analysis, we used the IMAGEJ Version 1.54p 17 February 2025 (Public Domain, BSD-2) program.

### 2.7. Biochemical Examination of Sciatic Nerve Tissue

Following death, sciatic nerve samples were excised and preserved at −20 °C until biochemical analyses were performed. A 10-mm distal nerve segment from the injury site was homogenized in phosphate-buffered saline (pH 7.4) with a glass homogenizer and centrifuged at 5000× *g* for 15 min. The supernatant was obtained, and total protein concentration was assessed utilizing the Bradford method, employing bovine serum albumin as the reference standard.

Nerve growth factor (NGF) and heat shock protein 70 (HSP-70) levels were measured utilizing commercially available enzyme-linked immunosorbent assay (ELISA) kits designed for rat proteins. Each sample was analyzed in duplicate according to the manufacturer’s guidelines, and absorbance values were recorded using a MultiscanGo microplate reader (Thermo Scientific Multiskan GO, Fisher Scientific Oy, Ratastie, Finland).

### 2.8. Assessment of Lipid Peroxidation (MDA Concentrations)

Malondialdehyde (MDA) concentrations in sciatic nerve tissues were measured to evaluate lipid peroxidation. Tissue homogenates were subjected to treatment with trichloroacetic acid and thiobarbituric acid reactive substances (TBARS) reagent, incubated at 100 °C for 60 min, subsequently cooled, and centrifuged at 3000 rpm for 20 min. The absorbance of the resultant supernatant was measured at 535 nm using a spectrophotometer, and MDA concentrations were ascertained from a standard curve established with tetraethoxypropane. The results were presented in nanomoles per gram of protein.

### 2.9. Statistical Analysis

Data were analyzed using Statistical Packages for Social Sciences 22.0 software (SPSS, IBM, Armonk, NY, USA). Categorical data are presented as frequency and percentages and analyzed using the Chi-square test. Measurement data are displayed as mean ± standard error of the mean (SEM), and the parametric component was compared using analysis of variance (ANOVA) and Student *t*-test. The non-parametric component was compared using the Mann-Whitney U test. Following a normality test, a Student *t*-test was used for comparison. *p* < 0.05 was considered statistically significant.

## 3. Results

### 3.1. Electrophysiological Evaluations

**Figure 2:**
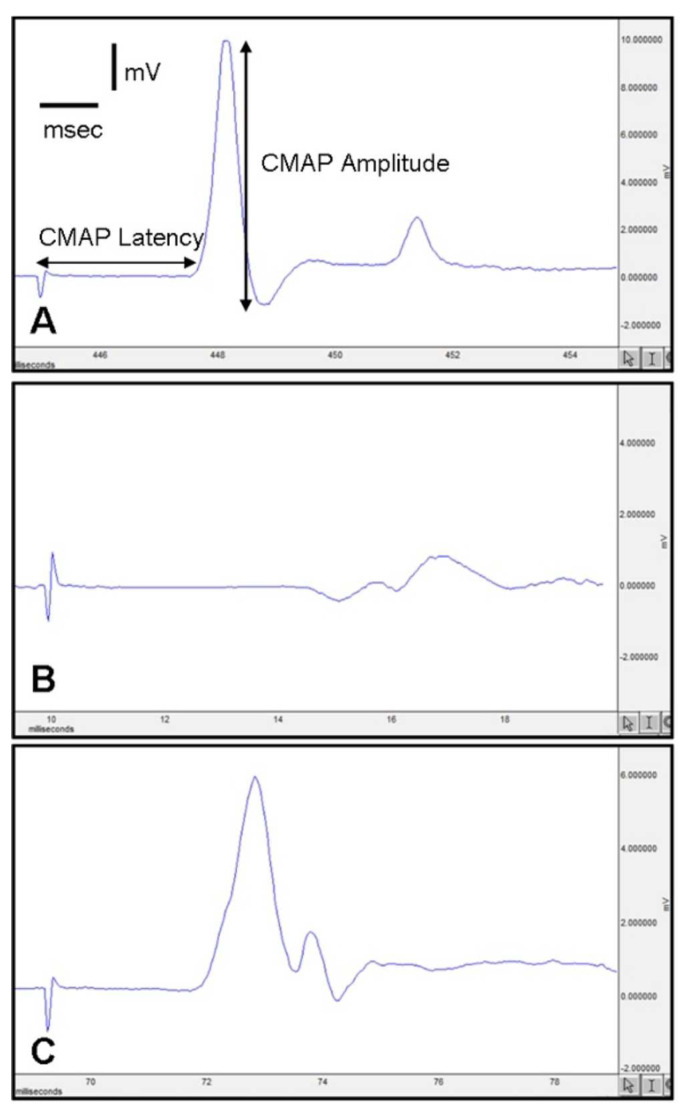
Electromyographic (EMG) evaluation: Representative waveforms and quantification of CMAP latency and amplitude in (**A**) Control, (**B**) Surgery + Saline (SS), and (**C**) Surgery + PEG (SPEG) groups.

Electromyographic recordings revealed significant differences between the groups. The Surgery and Saline (SS) group showed a prolonged CMAP latency (4.42 ± 0.2 ms) compared to the Normal Control (NC) group (2.28 ± 0.1 ms, *p* < 0.05). PEG 3350 treatment significantly reduced this latency to 3.3 ± 0.17 ms (*p* < 0.05 vs. SS). In terms of CMAP amplitude, the SS group showed a marked decrease (1.6 ± 0.2 mV) compared to the NC group (13.4 ± 0.9 mV, *p* < 0.01), while the PEG-treated group showed a significant increase in amplitude (4.9 ± 0.6 mV, *p* < 0.05 vs. SS) (Table 1, Figure 2).

### 3.2. Restoration of Functionality

The inclined plane test revealed compromised functional recovery post-surgery, with the SS group showing a markedly lower score (38.6 ± 8.1°) than the NC group (88.7 ± 2.5°, *p* < 0.01). The PEG 3350-treated (SPEG) cohort exhibited a significant enhancement (76.8 ± 6.5°) relative to the SS cohort (*p* < 0.01; Table 1).

### 3.3. Nerve Biochemical Examination

MDA concentrations in nerve tissue were markedly increased in the SS group (144.3 ± 4.5 nmol/g) relative to the NC group (82.2 ± 3.1 nmol/g, *p* < 0.05). PEG 3350 therapy markedly decreased MDA levels (95.6 ± 1.8 nmol/g) in comparison to the SS groups (*p* < 0.05). NGF levels were significantly reduced in the SS group (30.5 ± 1.2 pg/g) compared to the NC group (115.2 ± 1.9 pg/g, *p* < 0.05). PEG 3350 markedly elevated NGF levels (82.9 ± 1.5 pg/g) in comparison to the SS group (*p* < 0.01; Table 1). Moreover, HSP-70 levels were markedly increased in the SPEG group (10.1 ± 0.8 µg/mg) relative to the SS group (6.4 ± 0.5 µg/mg, *p* < 0.05; Table 2).

### 3.4. Histopathological and Immunohistochemical Observations

**Figure 3:**
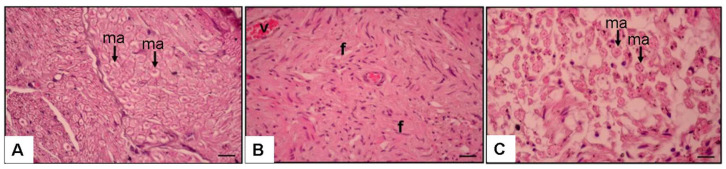
Magnification. Hematoxylin and Eosin × 20 (**A**) NC group. Myelin+Normal axon (ma) (arrow). (**B**) Surgery and Saline Group. Increased fibrosis (f), (**C**) SPEG group. Increased axon and myelin (ma).

**Figure 4:**
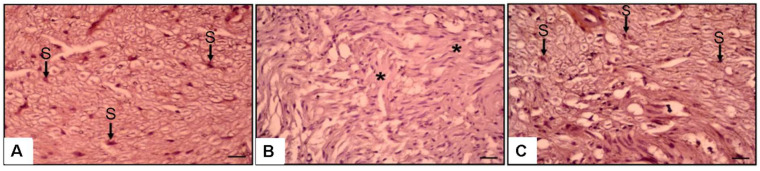
Magnification. NGF immunostaining. ×20 (**A**) NC group. Normal schwann cell (S). (**B**) Surgery and Saline Group. Very diminished axon, schwann cell and NGF immunoexpression (asterisk). (**C**) SPEG group. Increased schwann cell and NGF (S).

Hematoxylin, Eosin, and NGF immunostaining findings revealed significant histopathological variations across the groups (Figure 3 and Figure 4). The NC group had well-structured nerve fibers with normal axons and myelin sheath (Figure 3A).

**Figure 5:**
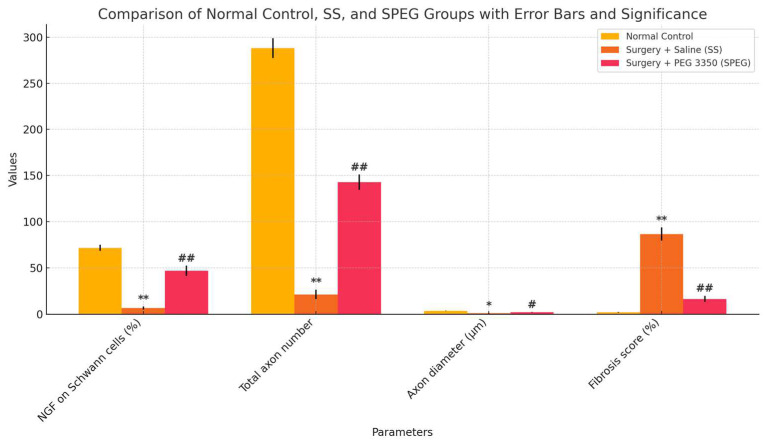
Data are expressed as mean ± SEM. * *p* < 0.05, ** *p* < 0.001 (different from control), # *p* < 0.01, ## *p* < 0.001 (different from SS Group).

Conversely, the SS group had significant fibrosis (86.5 ± 7.1%) (Figure 3B and Figure 5), markedly reduced axon counts (21.2 ± 5.1), and decreased Schwann cell and NGF immunoexpression (6.4 ± 1.7%) (Figure 4B and Figure 5).

The SP group exhibited substantial recovery, characterized by enhanced axonal structures (142.7 ± 8.3), Schwann cells, and NGF immunoexpression (46.7 ± 5.5%, *p* < 0.01) compared to the SS group (Figure 3C, Figure 4C and Figure 5). Fibrosis was markedly reduced in the SP group (16.3 ± 3.4%) relative to the SS group (*p* < 0.01) (Figure 3C, Figure 4C and Figure 5).

The axon diameter in the SS group was considerably reduced (0.9 ± 0.2 µm) compared to the NC group (3.48 ± 0.21 µm, *p* < 0.05). PEG 3350 treatment markedly enhanced axon diameter (2.04 ± 0.1 µm, *p* < 0.05; Figure 5). Histological and immunohistochemical assessments were performed to evaluate axon morphology and NGF immunoexpression. The results are shown in Figure 3, Figure 4 and Figure 5.

## 4. Discussion

Trauma-induced peripheral nerve injury is a significant contributor to global disability. Following a crush injury, an initial damage phase is followed by a prolonged secondary injury phase characterized by oxidative damage, inflammation, edema, and apoptosis. Experimental and clinical studies on PEG have shown neuroprotective effects [24]. Nevertheless, the mechanism by which PEG provides this protection remains ambiguous [14,25,26]. The anti-apoptotic, anti-inflammatory, and antioxidant effects are insufficiently defined.

The restorative results of peripheral nerve damage through spontaneous regeneration following injury are often insufficient, even after microsurgical procedures, especially in severe cases, requiring continued pharmacological investigation to alleviate the negative consequences of this condition [27]. The findings of this study demonstrate a multifaceted advantageous impact of PEG on peripheral nerve injury. The integration of improved electrophysiological measurements, axonal architecture, diminished fibrosis, lowered oxidative stress, and increased neurotrophic factor expression robustly endorses the potential therapeutic use of PEG in addressing peripheral nerve injuries.

In a study by Hibbard, they described facilitating the prompt fusion of severed axonal membranes after admission of PEG, thus reinstating axonal continuity and averting Wallerian degeneration [28]. This procedure facilitates swift reinnervation of distal targets and enhances behavioral rehabilitation in rats with sciatic nerve injuries. In a study by Zhou, similar to ours, PEG admission has demonstrated the ability to recover electrophysiological continuity and preserve myelin sheaths, which are essential for nerve function [29]. Everything showed us that the admission type, intraperitoneally or intraoperatively, may not change the healing effect.

The anti-inflammatory effects of PEG in many models, such as acute pancreatitis and acute lung injury, are known [30]. Likewise, PEG exhibited protective properties against systemic inflammation and sepsis by diminishing inflammatory cytokine expression and mortality rates [31]; however, in the study of Wang et al. They claim that PEG mitigated inflammation in the spinal cord injury model by stabilizing cellular membranes and diminishing calcium influx, thereby facilitating neuroprotection [32]. Moreover, the antioxidative effect of PEG was described in the spinal cord injury model [33]. In another study conducted by Kang et al., they claim that quercetin combined with PEG in a nanosuspension demonstrated significant antioxidant effects through the scavenging of reactive oxygen species (ROS) and reactive nitrogen species (RNS), thereby mitigating inflammation [34].

NGF promotes nerve regeneration via various mechanisms, such as activating autophagy in Schwann cells, modulating the NO-cGMP pathway, and processes reliant on RNA synthesis [35,36]. It functions synergistically with additional growth factors to augment nerve repair and possesses substantial neuroprotective properties [28]. Moreover, it facilitates axonal and myelin repair, improves functional recovery, and collaborates with other growth factors such as VEGF [33,37]. Its capacity to promote neurite outgrowth and collateral sprouting further highlights its therapeutic potential in nerve injury interventions [38]. In this study, in tissue biochemical analysis, NGF level shows extreme augmentation after PEG admission, which can accelerate nerve repair. Moreover, the augmentation of NGF immunoexpression on Schwann cells proves the reparative trigger effect of PEG on nerve cells.

HSP70 is recognized for its neuroprotective attributes, primarily due to its chaperone functions. HSP70 facilitates the correct folding of proteins and inhibits their collection, which is essential after nerve injuries [39]. HSP-70 may engage with proteins associated with cell death, consequently diminishing neuronal apoptosis and enhancing cell survival [39,40]. PEG and HSP-70 prevent ischemia-reperfusion injury (IRI) [41]. HSP-70 is a cytoprotective protein essential for cellular protection under stress conditions, including IRI. PEG enhances the expression of HSP-70, thereby aiding in the protection and recovery of cells through protein stabilization and repair of damaged proteins [41]. Although the study we just mentioned was performed for liver cells, we describe the cellular protection effect biochemically and histopathologically in nerve cells. According to these results, we can interpret that NGF supports the promoter, and HSP-70 supports the modulator function of HSP-70, and together, they produce a synergistic effect.

Existing techniques for inducing HSP70, including gene transfer and heat stress, are impractical for clinical application [39,42,43]. Consequently, current research aims to create pharmacological agents capable of safely and effectively inducing HSP-70 in humans. Weiss et al. state that PEG is thought to inhibit Wallerian degeneration by directly fusing the axolemma, the membrane encasing nerve axons, which is essential for nerve repair. During the initial 24 h following injury, no significant alterations in gene expression were noted. However, 918 differentially expressed genes (DEGs) were identified in the PEG-treated sciatic nerve in the subsequent four-week period, with a predominant 79% exhibiting upregulation [25]. The genes exhibited enrichment in pathways associated with nervous system development and growth, indicating improved nerve regeneration.

The dramatic increase in axon number and axon width, as well as the fibrosis score being in parallel with the histopathological findings of the study, are among the results that make our study strong. In this context, in addition to the previous studies, the fact that the levels of NGF and HSP-70 in sciatic nerve injury are in the direction of improvement after PEG application and also the increase in the levels of NGF in Schwan cells indicate that PEG causes improvement in nerve injury through HSP-70 as well as other mechanisms.

### Study Limitations

While this study demonstrates the beneficial effects of PEG 3350 in a rat model of sciatic nerve injury, the results may not fully translate to the complexity of human nerve regeneration. Although animal models are essential for preclinical research, further human studies are necessary to confirm these findings. The long-term effects and potential adverse outcomes of PEG therapy were not assessed. Future clinical trials are needed to evaluate human subjects’ safety, efficacy, and optimal treatment protocols.

## 5. Conclusions

The findings of this study indicate that PEG admission in cases of sciatic nerve damage significantly enhances and accelerates nerve regeneration. Subsequent research will clarify the specifics of this topic. Investigating the combined effects of mediators or pathways identified in treating peripheral nerve injury will optimize treatment outcomes. This situation should be considered in future studies.

## Figures and Tables

**Table 1:** Comparison of CMAP latency, CMAP amplitude, and inclined plane scores among groups. Data are presented as mean ± SEM. * *p* < 0.05, ** *p* < 0.01 (significantly different from control), # *p* < 0.05, ## *p* < 0.01 (significantly different from SS Group).

	Normal Control	Surgery and Saline (SS) Group	Surgery and PEG 3350 (SPEG) Group
EMG CMAP latency (ms)	2.28 ± 0.1	4.42 ± 0.2 *	3.3 ± 0.17 ##
EMG CMAP amplitude (mV)	13.4 ± 0.9	1.6 ± 0.2 **	4.9 ± 0.6 #
Inclaned plane score (°)	88.7 ± 2.5	38.6 ± 8.1 **	76.8 ± 6.5 ##

**Table 2:** Biochemical evaluation of sciatic nerve tissue. MDA, NGF, and HSP-70 levels across groups. Data are presented as mean ± SEM. * *p* < 0.05 (significantly different from control), # *p* < 0.05, ## *p* < 0.01 (significantly different from SS Group).

	Normal Control	Surgery and Saline (SS) Group	Surgery and PEG 3350 (SPEG) Group
Nevre MDA Level (nmol/g)	82.2 ± 3.1	144.3 ± 4.5 *	95.6 ± 1.8 #
Nerve NGF Level (pg/g)	115.2 ± 1.9	30.5 ± 1.2 *	82.9 ± 1.5 ##
Nerve HSP-70 Level (µg/mg)	5.6 ± 0.3	6.4 ± 0.5	10.1 ± 0.8 #

## Data Availability

The original contributions presented in the study are included in the article; further inquiries can be directed to the corresponding author.

## References

[1] Juckett L., Saffari T.M., Ormseth B., Senger J.-L., Moore A.M. (2022). The Effect of Electrical Stimulation on Nerve Regeneration Following Peripheral Nerve Injury. Biomolecules.

[2] Willand M.P., Nguyen M.-A., Borschel G.H., Gordon T. (2015). Electrical Stimulation to Promote Peripheral Nerve Regeneration. Neurorehabilit. Neural Repair.

[3] Padovano W.M., Dengler J., Patterson M.M., Yee A., Snyder-Warwick A.K., Wood M.D., Moore A.M., Mackinnon S.E. (2020). Incidence of Nerve Injury After Extremity Trauma in the United States. Hand.

[4] Acharya N., Acharya A.M., Bhat A.K., Upadhya D., Punja D., Suhani S. (2023). The outcome of polyethylene glycol fusion augmented by electrical stimulation in a delayed setting of nerve repair following neurotmesis in a rat model. Acta Neurochir..

[5] Mikesh M., Ghergherehchi C.L., Hastings R.L., Ali A., Rahesh S., Jagannath K., Sengelaub D.R., Trevino R.C., Jackson D.M., Bittner G.D. (2018). Polyethylene glycol solutions rapidly restore and maintain axonal continuity, neuromuscular structures, and behaviors lost after sciatic nerve transections in female rats. J. Neurosci. Res..

[6] Pan D., Mackinnon S.E., Wood M.D. (2020). Advances in the repair of segmental nerve injuries and trends in reconstruction. Muscle Nerve.

[7] Carvalho C.R., Reis R.L., Oliveira J.M. (2020). Fundamentals and Current Strategies for Peripheral Nerve Repair and Regeneration. Advances in Experimental Medicine and Biology.

[8] Van Nest D.S., Kahan D.M., Ilyas A.M. (2020). Polyethylene Glycol Fusion of Nerve Injuries: Review of the Technique and Clinical Applicability. J. Hand Microsurg..

[9] Zhao F.-Q., Jiang B.-G., Zhang P.-X., Zhang H.-B. (2006). The rule of proliferation after sciatic injury of rats: Immunohistological observation. Zhonghua Wai Ke Za Zhi.

[10] Pušnik L., Lechner L., Serša I., Cvetko E., Haas P., Jengojan S.A., Snoj Ž. (2024). 3D fascicular reconstruction of median and ulnar nerve: Initial experience and comparison between high-resolution ultrasound and MR microscopy. Eur. Radiol. Exp..

[11] Sąsiadek M.J., Szewczyk P., Bladowska J. (2012). Application of diffusion tensor imaging (DTI) in pathological changes of the spinal cord. Med. Sci. Monit..

[12] Alper A., Pashankar D.S. (2013). Polyethylene Glycol: A game-changer laxative for children. J. Pediatr. Gastroenterol. Nutr..

[13] Pasut G., Veronese F.M., Satchi-Fainaro R., Duncan R. (2006). PEGylation of proteins as tailored chemistry for optimized bioconjugates. Polymer Therapeutics I.

[14] Nemani S., Chaker S.B., Ismail H.M., Yao J., Chang M., Kang H., Desai M., Weikert D., Bhandari P.L., Drolet B. (2024). Polyethylene Glycol–Mediated Axonal Fusion Promotes Early Sensory Recovery after Digital Nerve Injury: A Randomized Clinical Trial. Plast. Reconstr. Surg..

[15] Britt J.M., Kane J.R., Spaeth C.S., Zuzek A., Robinson G.L., Gbanaglo M.Y., Estler C.J., Boydston E.A., Schallert T., Bittner G.D. (2010). Polyethylene Glycol Rapidly Restores Axonal Integrity and Improves the Rate of Motor Behavior Recovery After Sciatic Nerve Crush Injury. J. Neurophysiol..

[16] Bamba R., Waitayawinyu T., Nookala R.M., Riley D.C., Boyer R.B., Sexton K.W., Boonyasirikool C., Niempoog S., Kelm N.D., Does M.D. (2016). A novel therapy to promote axonal fusion in human digital nerves. J. Trauma Acute Care Surg..

[17] Sarac B.A., Wordsworth M., Schmucker R.W. (2024). Polyethylene Glycol Fusion and Nerve Repair Success: Practical Applications. J. Hand Surg. Glob. Online.

[18] Smith T.A., Zhou L., Ghergherehchi C.L., Mikesh M., Yang C.Z., Tucker H.O., Allgood J., Bushman J.S., Bittner G.D. (2024). Polyethylene glycol has immunoprotective effects on sciatic allografts, but behavioral recovery and graft tolerance require neurorrhaphy and axonal fusion. Neural Regen. Res..

[19] Sever I., Ozkul B., Atasoy O., Cini N., Bozkurt M., Erdogan M., Yaprak G., Erbas O. (2023). Neuroprotective effect of polyethylene Glycol (PEG-3350) on radiation-induced brain injury via membrane stabilization. Int. J. Radiat. Res..

[20] Luo J., Borgens R., Shi R. (2002). Polyethylene glycol immediately repairs neuronal membranes and inhibits free radical production after acute spinal cord injury. J. Neurochem..

[21] Ferrero-Andrés A., Panisello-Roselló A., Roselló-Catafau J., Folch-Puy E. (2020). Polyethylene glycol 35 ameliorates pancreatic inflammatory response in cerulein-induced acute pancreatitis in rats. World J. Gastroenterol..

[22] Siddiqui A.M., Cui X., Wu R., Dong W., Zhou M., Hu M., Simms H.H., Wang P. (2006). The anti-inflammatory effect of curcumin in an experimental model of sepsis is mediated by up-regulation of peroxisome proliferator-activated receptor-gamma. Crit Care Med..

[23] Chen H., Quick E., Leung G., Hamann K., Fu Y., Cheng J.-X., Shi R. (2009). Polyethylene Glycol Protects Injured Neuronal Mitochondria. Pathobiology.

[24] Rippee D.B., Glassman G.E., Chaker S.C., Assi P.E., Black J., Yao J., Pollins A.C., Thayer W.P. (2021). Polyethylene Glycol Treatment for Peripheral Nerve Repair in Preclinical Models. J. Neurol. Neuromed..

[25] Weiss S.N., Legato J.M., Liu Y., Vaccaro C.N., Da Silva R.P., Miskiel S., Gilbert G.V., Hakonarson H., Fuller D.A., Buono R.J. (2024). An analysis of differential gene expression in peripheral nerve and muscle utilizing RNA sequencing after polyethylene glycol nerve fusion in a rat sciatic nerve injury model. PLoS ONE.

[26] Paskal A.M., Paskal W., Pietruski P., Wlodarski P.K. (2019). Polyethylene Glycol: The Future of Posttraumatic Nerve Repair? Systemic Review. Int. J. Mol. Sci..

[27] Yalçın M.B., Bora E.S., Erbaş O. (2024). The Effect of Liraglutide on Axon Regeneration and Functional Recovery after Peripheral Nerve Lesion. Curr. Issues Mol. Biol..

[28] Hibbard E.A., Sengelaub D.R. (2022). Intraneural Topography of Rat Sciatic Axons: Implications for Polyethylene Glycol Fusion Peripheral Nerve Repair. Front. Cell. Neurosci..

[29] Zhou L., Venkudusamy K., Hibbard E.A., Montoya Y., Olivarez A., Yang C.Z., Leung A., Gokhale V., Periyasamy G., Pathak Z. (2024). Polyethylene glycol fusion repair of severed sciatic nerves accelerates recovery of nociceptive sensory perceptions in male and female rats of different strains. Neural Regen. Res..

[30] Ferrero-Andrés A., Panisello-Roselló A., Serafín A., Roselló-Catafau J., Folch-Puy E. (2020). Polyethylene Glycol 35 (PEG35) Protects against Inflammation in Experimental Acute Necrotizing Pancreatitis and Associated Lung Injury. Int. J. Mol. Sci..

[31] Ackland G.L.P., Del Arroyo A.G., Yao S.T., Stephens R.C.F., Dyson A.M., Klein N.J.P., Singer M.M., Gourine A.V. (2010). Low-molecular-weight polyethylene glycol improves survival in experimental sepsis. Crit. Care Med..

[32] Wang X.-J., Shu G.-F., Xu X.-L., Peng C.-H., Lu C.-Y., Cheng X.-Y., Luo X.-C., Li J., Qi J., Kang X.-Q. (2019). Combinational protective therapy for spinal cord injury medicated by sialic acid-driven and polyethylene glycol based micelles. Biomaterials.

[33] Luo J., Borgens R., Shi R. (2004). Polyethylene Glycol Improves Function and Reduces Oxidative Stress in Synaptosomal Preparations following Spinal Cord Injury. J. Neurotrauma.

[34] Kang S.G., Lee G.B., Vinayagam R., Do G.S., Oh S.Y., Yang S.J., Kwon J.B., Singh M. (2022). Anti-Inflammatory, Antioxidative, and Nitric Oxide-Scavenging Activities of a Quercetin Nanosuspension with Polyethylene Glycol in LPS-Induced RAW 264.7 Macrophages. Molecules.

[35] Li R., Li D., Wu C., Ye L., Wu Y., Yuan Y., Yang S., Xie L., Mao Y., Jiang T. (2020). Nerve growth factor activates autophagy in Schwann cells to enhance myelin debris clearance and to expedite nerve regeneration. Theranostics.

[36] Burstein D.E., Greene L.A. (1978). Evidence for RNA synthesis-dependent and -independent pathways in stimulation of neurite outgrowth by nerve growth factor. Proc. Natl. Acad. Sci. USA.

[37] Xia B., Lv Y. (2018). Dual-delivery of VEGF and NGF by emulsion electrospun nanofibrous scaffold for peripheral nerve regeneration. Mater. Sci. Eng. C.

[38] Diamond J., Coughlin M., Macintyre L., Holmes M., Visheau B. (1987). Evidence that endogenous beta nerve growth factor is responsible for the collateral sprouting, but not the regeneration, of nociceptive axons in adult rats. Proc. Natl. Acad. Sci. USA.

[39] Kim J.Y., Barua S., Huang M.Y., Park J., Yenari M.A., Lee J.E. (2020). Heat Shock Protein 70 (HSP70) Induction: Chaperonotherapy for Neuroprotection after Brain Injury. Cells.

[40] Margiana R., Salma N.M., Jusuf A.A. (2024). Impact of Platelet-Rich Plasma on Sciatic Nerve Injury in Rats: HSP 70 Expression and Histological Changes in the Anterior Horn of the Spinal Cord. South East. Eur. J. Public Health.

[41] Zaouali M.A., Bejaoui M., Calvo M., Folch-Puy E., Pantazi E., Pasut G., Rimola A., Ben Abdennebi H., Adam R., Roselló-Catafau J. (2014). Polyethylene glycol rinse solution: An effective way to prevent ischemia-reperfusion injury. World J. Gastroenterol..

[42] Yenari M.A., Giffard R.G., Sapolsky R.M., Steinberg G.K. (1999). The neuroprotective potential of heat shock protein 70 (HSP70). Mol. Med. Today.

[43] Yalçın M.B., Bora E.S., Erdoğan M.A., Çakır A., Erbaş O. (2023). The Effect of Adipose-Derived Mesenchymal Stem Cells on Peripheral Nerve Damage in a Rodent Model. J. Clin. Med..

